# Key performance indicators in emergency department simulation: a scoping review

**DOI:** 10.1186/s13049-024-01318-7

**Published:** 2025-01-30

**Authors:** Mohammad Hossein Mehrolhassani, Anahita Behzadi, Elaheh Asadipour

**Affiliations:** Health Services Management Research Center, Institute for Futures Studies in Health, Kerman University of Medical Sciences, Kerman, Iran

**Keywords:** Key performance indicators (KPIs), Emergency department, Simulation

## Abstract

**Background:**

One way to measure emergency department (ED) performance is using key performance indicators (KPIs). Thus, identifying reliable KPIs can be critical in appraising ED performance. This study aims to introduce and classify the KPIs related to ED in simulations through the Balanced Scorecard (BSC) framework.

**Method:**

This scoping review was performed in 2024 without any time limitation based on the Arksey and O'Malley framework. The electronic databases of PubMed, Scopus, Web of Science, EMBASE, MathSciNet, Google Scholar, and Persian databases such as IranDoc, MagIran, and SID were searched. The winter simulation conference was also investigated through manual searching. Furthermore, the screening process of included studies was based on the PRISMA reporting checklist. The data were analyzed by content analysis deductively and inductively. The extracted KPIs were coded as analysis units and transferred to the MAXQDA2020 software. Then, the KPIs were integrated and organized based on similarity. Moreover, the two authors discussed disagreements to reach a consensus on the final codes. The final KPIs classification was carried out based on the BSC framework to achieve a holistic view. The BSC is a managerial tool for evaluating organizations' performance via different dimensions. It contains four main dimensions: Customer, Financial, Growth and infrastructure, and Internal Processes. In addition, the management (vision, objectives, and strategies) has been positioned at the heart of the framework.

**Result:**

Initially, 4257 articles were retrieved, and 125 articles were included after screening. Finally, 109 KPIs were extracted and classified into five categories. They include input, processing time, cost and revenue, utilization and productivity, and output indicators. Then, each category of KPIs was positioned in the BSC framework dimensions. Additionally, the findings showed that most indicators were related to the time of process indicators.

**Conclusions:**

The study findings have collected a comprehensive set of KPIs to measure ED performance in simulations. These results can assist policymakers, managers, and researchers in measuring ED performance and help improve ED performance through a holistic view.

**Supplementary Information:**

The online version contains supplementary material available at 10.1186/s13049-024-01318-7.

## Background

Emergency departments (EDs) are hospital patients' first contact. The efficiency of ED performance affects hospital functions and treatment outcomes. Meanwhile, The ED's complexity makes managing them harder [1]. From another point of view, identifying the strengths and weaknesses of ED performance and comparing ED departments can improve the quality of care and responsiveness [2]. Therefore, ED performance evaluation prepares managers to respond to the challenges effectively. Additionally, performance indicators help ED managers identify the operations that should be improved and pursue the appropriate strategy to cope with the sudden environmental transformation [3].

Measuring ED performance can be beneficial by improving or removing non-value-added procedures. Thus, Identifying the transparent, reliable, achievable, appropriate, and exact key performance indicators (KPIs) can be the first step in appraising ED performance [4].

The simulation methods are suitable for modeling ED's complexity and stochastic nature. In other words, simulation is a method that implements real system behaviors in detail. This is a proper technique to measure ED performance [5]. It provides the possibility to reduce the costs and risks of the practical implementation of the solutions [6]. The concurrent effect of various scenarios regarding several ED performance indicators is analyzed in a simulation model.

The system operation is measured according to the simulation model output, and the simulation output's accuracy depends on its input's precision. Moreover, the defined KPIs are an important input for evaluating ED operations and scenarios. Therefore, the high quality of ED performance measurement relies on the suitable collection of KPIs [5].

Previously, measuring organizational performance was based on financial indicators. They mainly concentrated on short-term goals and ignored long-term goals such as investment in the future and creating values. So, a comprehensive performance measurement requires something beyond financial indicators. A Balanced Scorecard (BSC) will complete financial indicators with the predictors of future performance indicators [7]. Evaluating ED performance requires a set of indicators that cover all critical dimensions of performance measurement. BSC is a managerial technique and performance measuring system that provides a wide and bright perspective in determining the strengths and weaknesses of organizational performance [8].

There is a lot of simulation literature on measuring ED performance. Some performance indicators are taken into account in simulation papers frequently, however some are rarely applied. Thus, it is caused to ignore some dimensions of ED performance evaluation. The BSC framework could evaluate performance from various dimensions: growth and infrastructure, customer, financial, and internal processes, and considering management (Vision, Objectives, and Strategies) at the center of the framework [9]. Consequently, organizing the extracted KPIs according to the BSC framework would complete the ED performance measurement puzzle. As far as we know, no comprehensive collection and organization of KPIs can be used to measure ED performance in simulations. A scoping review was required to determine the KPIs of ED in recently published simulation studies, which could shed light for administrators and operational researchers to find out the neglected aspects of ED performance measurement in simulation studies. Thus, this scoping review aimed to identify and classify the KPIs of ED in simulation studies using the BSC framework.

## Methods

This present study is a scoping review conducted based on the Arksey and O'Malley framework. It describes the scoping review in five steps: (1) Choosing the main research question (2) Determining related studies (3) Study selection (4) Charting the data (5) Collating, summarizing, and reporting the results [10].

### Choosing the research question

In this paper, we have attempted to examine the simulation literature to extract the emergency department's KPIs. Then, we have classified the KPIs based on the BSC framework to reach an organized collection of ED performance indicators applicable in modeling and simulation. A scoping review could be a proper option for the present study since a broad spectrum of KPIs in ED has been explored without focusing on details. Furthermore, we aimed to identify which KPIs and performance measurement aspects in ED simulation require further investigations. In a scoping review study, article selection doesn't have rigid criteria and is more convenient compared to systematic reviews. Therefore, it can include a wide range of simulation studies. This could be useful in complicated issues such as simulation in ED.

The leading question of this scoping review is, "What are the Emergency Department's KPIs in simulation studies?".

In this scoping review two objectives have been followed:

1. Identifying the KPIs, that have been used in the performance measurement of ED simulation studies.
2. Classifying the extracted KPIs based on the BSC framework to prepare a comprehensive set of the KPIs of ED.

### Determining related studies

The study follows the Preferred Reporting Items with the Systematic Review and Meta-analysis Statement (PRISMA) reporting checklist [11]. The electronic databases and primary relevant journals were investigated in this scoping review. The databases of PubMed, Scopus, Web of Science, EMBASE, MathSciNet, Google Scholar, and Persian databases of MagIran, IranDoc, and SID to identify ED simulation-based literature relevant to the KPIs were examined. The main part of the search was carried out in 2024 From September to November without any time limitation. Furthermore, a manual search was conducted on the Winter Simulation Conference (WSC) archive as a source of simulation papers from 2000 to 2023. Meanwhile, the references of retrieved studies were reviewed to increase comprehensiveness. The keywords that were used consisted of Medical Subject Headings (MeSH) and common related keywords as follows (Table 1).

**Table 1:** Search strategy

PCC	Keywords	Search terms
Population	Simulation	“System analysis” OR “simulation” OR “system dynamics analysis” OR “agent-based modeling”
Concept	Key Performance Indicators (KPIs)	“Key performance indicator” OR “performance metric” OR “process measure” OR “workflow” OR “patient flow “OR “Quality indicator” OR “organizational efficiency”
Context	Emergency Department (ED)	” Emergency department” OR “emergency medical service” OR “hospital emergency department” OR “emergency ward” OR “emergency room”

Moreover, the eligibility criteria of the retrieved studies were included in Table 2.

**Table 2:** Inclusion and exclusion criteria

*Inclusion Criteria*	
1. Full-text available papers	
2. Papers in the hospital emergency department, not other department	
3. Papers that were published in Persian or English language	
4. Papers that evaluate emergency department performance	
*Exclusion Criteria*	
1. The conference papers except for the WSC conference, letters to the editor, editorials, commentaries, clinical trials, books, serials, and opinion articles	
2. Papers that were done simulation with the teaching purposes	
3. Papers that describe the simulation of clinical and physiological procedures	
4. Papers were about the management of emergencies and crises	
5. Paper associated with prehospital emergency medical service (EMS)	

A proficient librarian prepared the primary search strategy, and research team members carried out more editions. To ensure sensitivity and specificity used synonyms with the Boolean operators “OR” and “AND”. The complete search string of electronic databases can be found in the appendix file. In addition, retrieved studies were transferred to EndNote citation management software (version X9) to find duplicates.

#### Selecting the studies

Two authors independently reviewed the retrieved studies based on inclusion and exclusion criteria. It can reduce bias in the study selection process. Firstly, the title and abstract were examined, and irrelevant studies were removed through a preliminary screening. Secondly, the full text of the studies that passed the initial evaluation was achieved to investigate precisely according to the inclusion and exclusion criteria. Then, the final studies were selected based on their consideration of key performance indicators in ED simulation-based studies. Furthermore, any disagreement on the study selection was resolved through discussion with the research team members. Considering that, the quality assessment of articles in scoping reviews is not usual, the quality of the articles was not examined in this study.

#### Charting the data

Two reviewers carried out the data extraction considering the research question. A comprehensive data extraction form was jointly designed by two reviewers in Microsoft Excel 2019 software and tested randomly on 10 included papers. Information related to the characteristics of selected studies and simulations was transformed into the designed Excel form. The form contains key variables such as Title, authors, research type, methodology, country, simulation method, and software. Moreover, the common key performance indicators related to the ED were extracted and imported to the MAXQDA 2020 software to define the theme and sub-themes. One author extracted data from included studies and the other one evaluated the data. They resolved any disagreement on data extraction through consensus discussion and negotiation with the third author.

#### Collating, summarizing, and reporting the results

Two authors analyzed the extracted data independently to address the research question and objectives. The content analysis was used to analyze the extracted data. content analysis is a systematic way of document analysis. Furthermore, the deductive and inductive approaches are two main content analysis strategies [12]. In the present study, data analysis was performed through both deductive and inductive approaches. Initially, the papers were studied several times to find key performance indicators related to the ED, inductively. The identified KPIs were coded as units of analysis and transferred to the MAXQDA2020 software. The homogeneous KPIs were integrated and categorized based on similarity. Two authors reiterated this process to reach a consensus on the final codes. Then, the BSC framework was selected to organize the refined KPIs. It is a famous model and an effective tool to measure performance. It can ensure that various aspects are included in the performance measurement process [13]. The suggested BSC model contains four main dimensions: Customer, Financial, Growth and infrastructure, and Internal Processes. In addition, the management (Vision, Objectives, and Strategies) has been positioned at the heart of the framework [9]. The BSC dimensions can include all aspects of ED performance measurement and are suitable for organizing KPIs in sophisticated environments such as ED. This framework considers financial and nonfinancial indicators simultaneously. On the other hand, implementing a valid simulation model of ED requires a comprehensive performance assessment. So, utilizing BSC makes it possible to measure the different dimensions of ED performance. Moreover, the BSC framework facilitates identifying KPIs that have high value in measuring ED performance, and align with the ED's goals [14].

In this stage, the KPIs related to every dimension of the BSC model were grouped, deductively. After that, the theme and sub-theme were created. They were reviewed, edited, and labeled. Finally, a comprehensive balance set of KPIs related to the ED simulation-based studies was identified and organized according to the BSC framework. It is worth noting that two authors performed the data analysis procedures, and disagreements were referred to the third author.

## Results

**Fig. 1:**
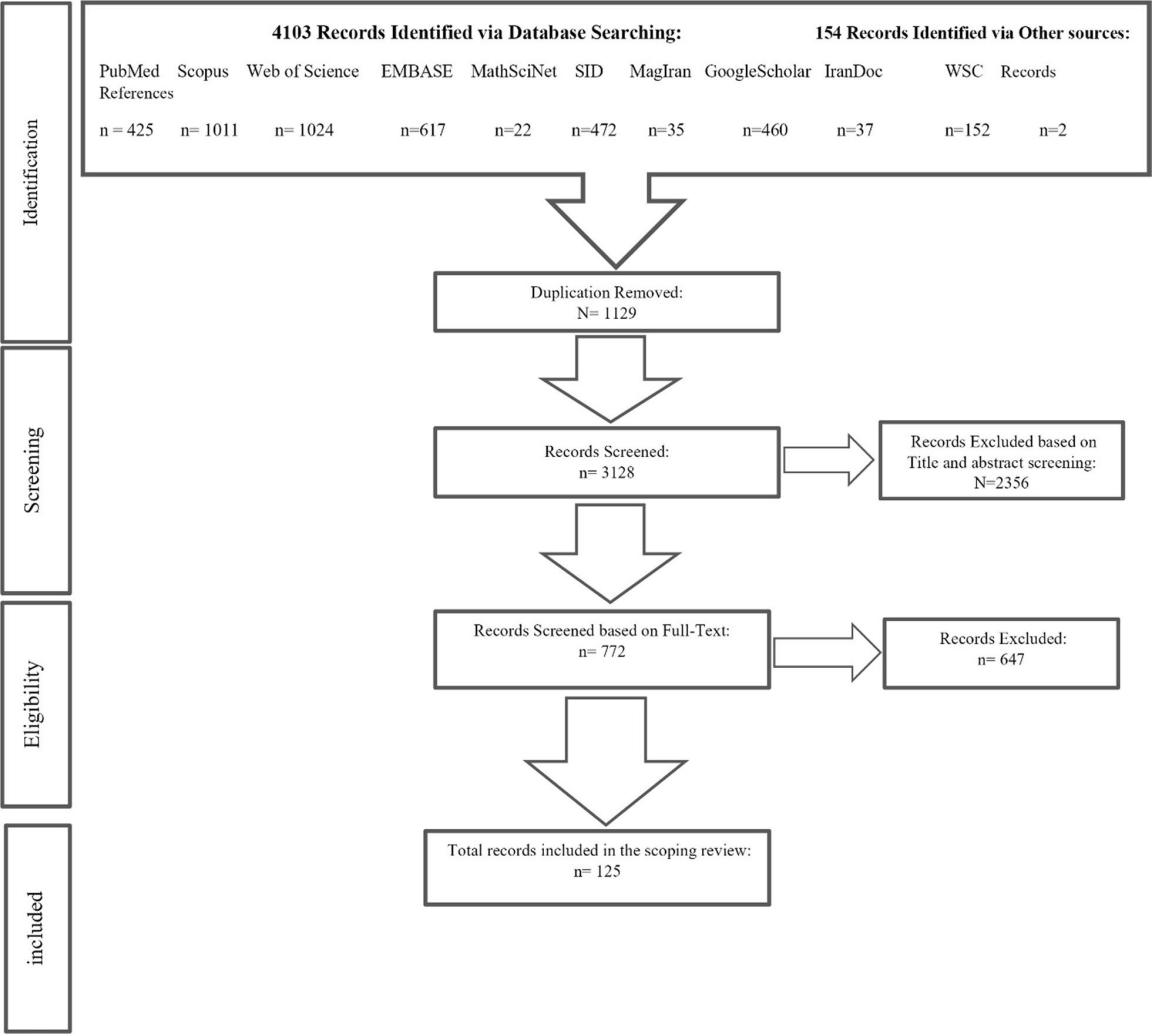
The flow chart of selecting relevant papers based on PRISMA

Our search initially retrieved 4257 studies, and after excluding 1129 duplicate studies, we were left with 3128. These were reviewed based on their title and abstract, leading to the exclusion of 2356. The full text of 772 studies was then evaluated for relevancy based on our inclusion and exclusion criteria, excluding 647 articles. Finally, we included 125 significant articles in our study. Figure 1 illustrates the PRISMA and details the selection of articles.

**Fig. 2:**
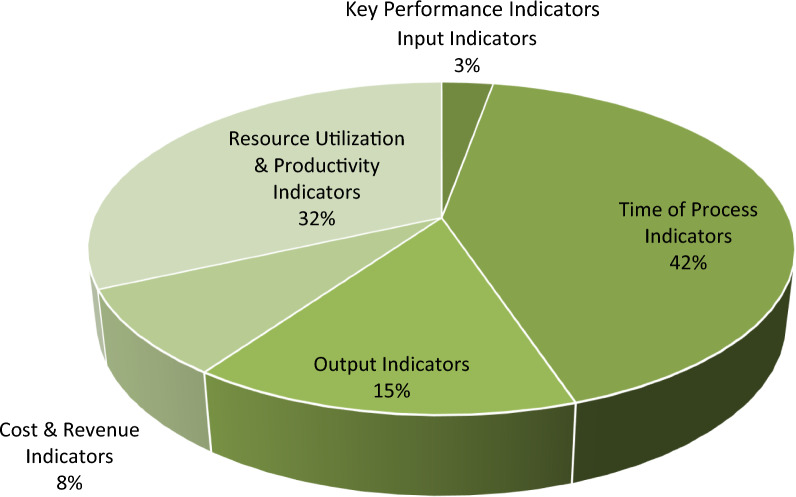
The distribution of ED KPIs

These indicators were then classified based on the BSC, resulting in the identification of 109 KPIs. These were further categorized into five main groups: input indicators (N = 3), process time indicators (N = 46), output indicators (N = 16), cost and revenue indicators (N = 9), and resource utilization and productivity indicators (N = 35). The distribution of these ED KPIs is presented in Fig. 2.

**Fig. 3:**
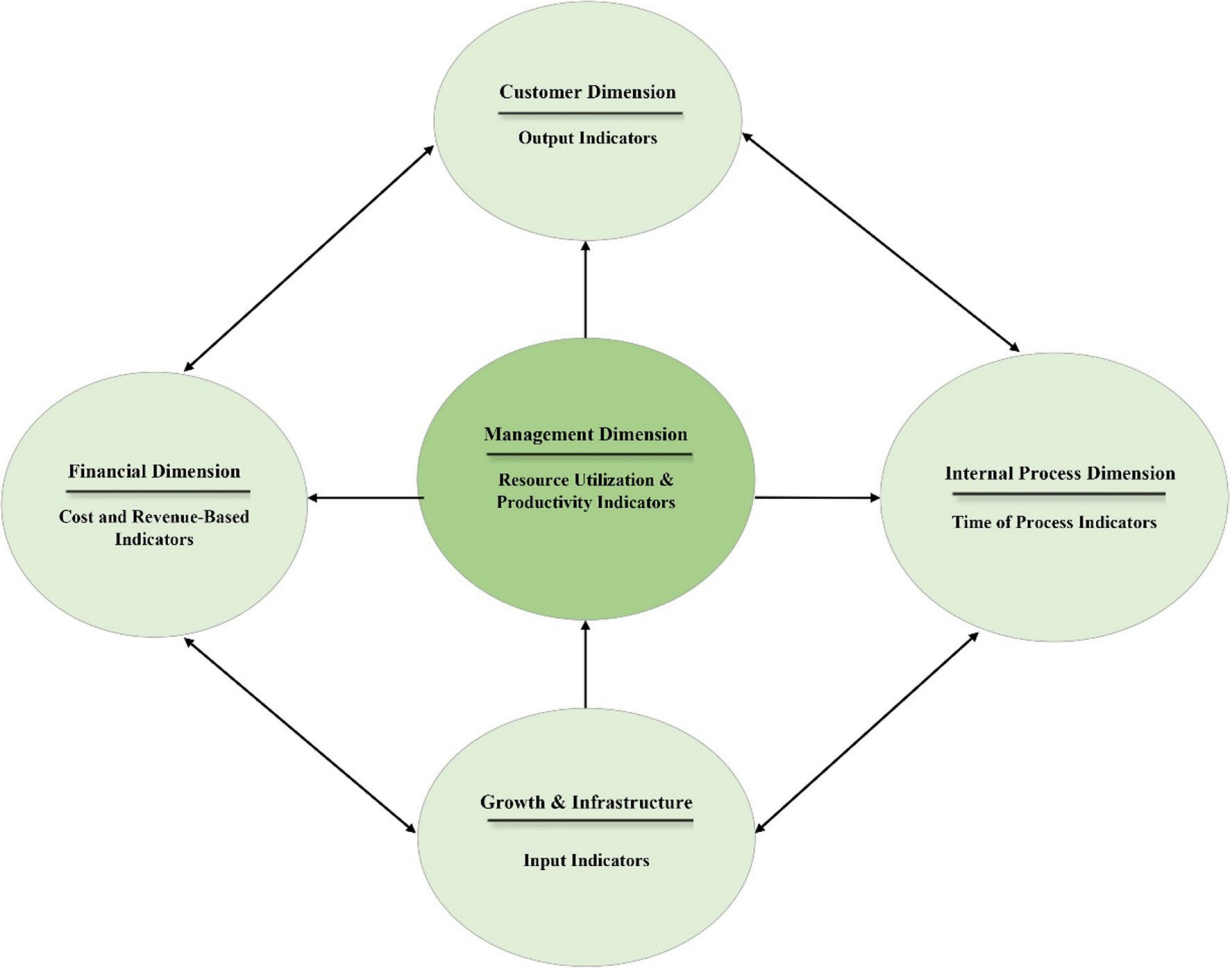
Classifying the emergency department KPIs based on BSC

Each category of indicators was classified according to the comprehensive BSC framework. As can be seen in Fig. 3, the management dimension is placed in the center. Other dimensions such as the customer dimension, internal processes, growth and infrastructure, and finance are placed around. The placement of key performance indicators in the BSC framework are as follows Resource utilization and productivity indicators were inserted in the management dimension, input indicators in the growth and infrastructure dimension, output indicators in the customer dimension, process time indicators in the internal processes dimension, and cost and revenue indicators in the financial dimension (Fig. 3).

As Table 3 demonstrates, 40% of retrieved papers were published from 2007 to 2016, and 54.4% were published from 2017 to 2024. 82.4% of studies used mixed methods as a research method. More than 56.8% of the studies used DES as a simulation method, and the most used software is Arena (37.6%), Simul8 (8%), and Net Logo (5.6%).

**Table 3:** The studies characteristics

Information		NO, %	References
Year	1986–1996	N = 1, (0.8%)	[47]
	1997–2006	N = 6, (4.8%)	[41, 48–52]
	2007–2016	N = 50, (40%)	[7, 8, 13, 18, 21, 22, 24, 25, 34, 36, 40, 44, 45, 53–89]
	2017–2024	N = 68, (54.4%)	[1, 5, 20, 23, 26, 27, 29, 31, 35, 37–39, 42, 43, 90–143]
Research Type	Quantitative	N = 10, (8%)	[7, 8, 27, 36, 56, 58, 87, 112, 115, 120]
	Mix Method	N = 103, (82.4%)	[1, 13, 20–26, 31, 34, 35, 37–45, 49–52, 54, 55, 57, 59–63, 65–75, 77–86, 88–93, 95–98, 100–107, 109–111, 113, 114, 117–119, 121–143]
	Review papers	N = 9, (7.2%)	[5, 18, 29, 53, 64, 76, 94, 99, 116]
	NA*	N = 3, (2.4%)	[47, 48, 108]
Country	Iran	N = 30, (24%)	[7, 8, 25–27, 31, 34, 37, 40, 54–57, 75, 85, 86, 90–92, 98, 101, 120–122, 124, 126, 128, 142, 143]
	USA, Canada	N = 25, (20%)	[41, 47–49, 60, 61, 63, 65, 66, 68–70, 74, 77, 87, 89, 105, 106, 111, 114, 123, 131, 134, 138, 141]
	UK, France, Germany, Irland, Italy, Norway, Spain, Sweden, Switzerland	N = 28, (22.4%)	[1, 13, 21, 29, 44, 52, 83, 84, 93, 96, 97, 100, 102, 103, 109, 110, 113, 115, 117–119, 125, 129, 132, 135–137, 139]
	Taiwan, Hongkon, Singapore, South Korea	N = 6, (4.8%)	[38, 58, 60, 79, 81, 127]
	Brazil, Latin America	N = 3, (2.4%)	[23, 95, 108]
	Egypt, Jordan, Kuwait, UAE, Turkey	N = 10, (8%)	[18, 20, 24, 42, 73, 88, 104, 107, 133, 140]
	NA	N = 23, (18.4%)	[5, 22, 35, 37, 39, 43, 45, 50, 51, 53, 62, 64, 67, 71, 72, 76, 78, 82, 94, 99, 112, 116, 130]
Simulation Method	DES	N = 71, (56.8%)	[7, 8, 20, 24, 25, 27, 31, 34, 36, 38–40, 42, 45, 50, 52, 54–56, 60, 62, 63, 65–67, 70, 71, 73, 77–81, 83, 85–90, 93, 96–98, 101, 103–107, 113–115, 117–119, 121–124, 128–130, 133, 134, 136–141]
	ABS	N = 11, (8.8%)	[21, 23, 69, 76, 82, 84, 95, 102, 109, 111, 125]
	Hybrid	N = 9, (7.2%)	[1, 26, 35, 108, 110, 120, 127, 132, 135]
	DS	N = 1, (0.8%)	[22]
	Monte Carlo, Slam	N = 2, (1.6%)	[47, 68]
	NA	N = 31, (24.8%)	[5, 13, 18, 29, 37, 41, 43, 44, 48, 49, 51, 53, 57–60, 61, 64, 72, 74, 75, 91, 92, 94, 99, 100, 112, 116, 126, 131, 142, 143]
Software	Arena	N = 47, (37.6%)	[8, 25–27, 34, 36–38, 40, 41, 43–45, 50, 51, 54–56, 60, 61, 63, 66, 70, 75, 77, 83, 85, 89–91, 98, 101, 105, 106, 113, 117, 121–124, 126, 128, 130, 133, 137, 138, 142, 143]
	Simul8	N = 10, (8%)	[42, 57, 58, 60, 65, 79, 103, 104, 114, 129]
	Net Logo	N = 7, (5.6%)	[21, 23, 84, 95, 109, 111, 125]
	Anylogic	N = 10, (8%)	[1, 35, 78, 93, 110, 127, 131, 135, 139, 140]
	SIMIO	N = 3, (2.4%)	[39, 80, 87]
	Flexim	N = 2, (2.4%)	[97, 108]
	Matlab	N = 2, (2.4%)	[62, 102]
	Med Model	N = 2, (2.4%)	[49, 68]
	Process Model	N = 2, (2.4%)	[67, 107]
	CPN Tools	N = 1, (0.8%)	[7]
	eM-plant	N = 1, (0.8%)	[60]
	Extent, Microsoft Access	N = 1, (0.8%)	[48]
	JADE	N = 1, (0.8%)	[119]
	Micro Saint Sharp	N = 1, (0.8%)	[52]
	Service model	N = 1, (0.8%)	[73]
	Simiscrip	N = 1, (0.8%)	[24]
	Simmer	N = 1, (0.8%)	[115]
	SimProcess	N = 1, (0.8%)	[53]
	Visual Paradigm	N = 1, (0.8%)	[92]
	Hybrid (Arena + NetLogo Arena + MATLAB Arena + Minitab + Enterprise Dynamics (ED) Simul8 + R + Excel Flexim + Minitab)	N = 5, (4%)	[20, 31, 86, 96, 120]
	NA	N = 25, (20%)	[5, 13, 18, 22, 29, 47, 53, 64, 69, 71, 72, 76, 82, 88, 94, 99, 100, 112, 116, 118, 132, 134, 136, 141, 143]

*NA Not Assign

Table 4 shows that 'human resource indicators,' 'physical spaces,' and 'facilities and equipment' are the three leading input indicators. The human resource indicator, being the most replicated among the input indicators, is a key factor in program success. The distribution of input indicators in retrieved studies indicates that 30% of papers discussed human resources, 17% focused on facility and equipment, and 10% argued the importance of physical spaces.

**Table 4:** Input-related Emergency Department KPIs

Theme	Sub-theme	Code	Consideration	References
input-related indicators	1. Human Resource	Physicians/Doctors, Nurses, Diagnostic Personnel, Pharmacist, Technicians, Delegate, Secretary/Clerk, Receptionist, Administrative Staff	Number	[20, 21, 23, 35, 40, 44, 50–52, 54, 58–60, 63, 69, 76, 78, 79, 82, 84, 86, 92, 93, 95–97, 102, 107, 109, 111, 112, 125, 127, 130]
	2. Physical Spaces (ED)	Pediatrics, Medicine, Orthopedics, Triage rooms, CPR, Reception, Patient Room, ED treatment locations, Chest Pain Unit (CPU), Radiology, LAB, Injection Room, Exam Rooms, ED Specialist room, General Physician room	Square meters, location, and distance	[20, 34, 35, 51, 60, 76, 86, 97, 102, 118, 124]
	3. Facilities and equipment	Ambulances, Registration Desk, care box, medical devices, ECG, Trolleys, Stretchers, Beds, Workstation, Radiology facilities, Surgery facilities	Number	[40, 44, 50, 54, 58, 60, 69, 82, 86, 92, 93, 96, 97, 109, 112, 114, 118, 125]

Forty-six indicators related to process time were obtained by reviewing this study's articles, and Table 5 shows that these indicators are classified into three main categories. These three main categories are waiting times (WTs), time intervals of services, and time spent on services. Seventeen indicators were included in the "waiting time" category, 15 indicators were included in the "time intervals of services" category and 14 indicators were included in the "time spent on services" category. Then, each category classified the indicators based on the type of service (Admission, Triage, treatment, diagnostic procedures, assignment, and patient discharge). It seems "Waiting Time" is one of the most critical "Process Time" indicators and the most replicated among the time indicators in the reviewed studies. Also, the "Throughput time" and "Waiting Count" are placed in the subsequent ranks.

**Table 5:** Processing Time-related Emergency Department KPIs

Theme	Sub-Theme	Code		Consideration	References
Time of Process-related indicators	Waiting Time (WT)	1. Overall Waiting Time (WT)		Holding patient Time	[8, 13, 18, 20, 23–27, 31, 34–38, 40, 45, 50, 54–60, 64, 71, 75, 77, 86, 88, 89, 91, 92, 94, 98, 99, 101, 106, 107, 112, 113, 116, 119, 120, 122, 124, 126, 128, 133, 134, 142]
		Triage and Admission	2. Waiting time to triage/Register	Avg	[13, 18, 64, 70]
			3. Waiting time before admission to the consultation room	Avg	[64]
			4. Waiting Time (triage to starting of visit)	Sum	[113]
		Treatment	5. Waiting Time (from Arrival until evaluation by a practitioner)	Sum	[102, 104, 105, 137]
			6. Wait for bed	Sum	[18, 45, 64]
			7. Waiting time for diagnostic/treatment	Avg	[18, 63]
			8. Waiting time to the doctor	Avg	[1, 18, 47, 56, 63]
			9. Waiting to be seen (WTBS)	Sum	[69]
			10. Waiting in nurse > 20 min	Cut of point	[1]
		Diagnosis	11. Waiting time for test result	Avg	[13, 45]
		Discharge and Disposition	12. Boarding Time	Sum	[5, 13, 56, 71, 93, 136]
			13. Waiting time to discharge	Avg	[13, 18]
		Resource and Space	14. Wait time for each resource	Avg	[36, 41]
			15. Waiting time in the queue	Avg	[7, 61, 64, 90, 121, 130]
			16. Waiting time in the waiting room	Sum	[1, 71]
			17. Buffer/wasting Time	Sum	[45, 64, 89]
	Time Interval of Services	1. The time interval of the patient throughout the process		Sum	[41]
		Triage and Admission	2. The time between arrival and triage	Sum	[64, 74, 140]
			3. Triage to disposal decision time	Sum	[79]
			4. The time between triage and registration	Sum	[64]
			5. Triage to Bedtime	Sum	[64, 87, 89]
			6. DOT (difference between the starting time of triage to visit)	Sum	[115]
			7. Time from registration to ED physician Consult and to discharge	Sum	[64]
		Treatment	8. Door to bed	Sum	[45, 63, 66]
			9. Time until first seen	Sum	[18, 75, 80, 114]
			10. Time to doctor	Sum	[45, 112]
			11. Door to doctor time	Sum	[5, 44, 66, 74, 79, 93, 117, 140, 143]
			12. Doctor to Disposition	Sum	[45, 115]
			13. Time to treatment (TTT)	Sum	[1, 80, 132, 135]
		Discharge and Disposition	14. Disposition to door out	Sum	[45]
			15. First assessment to discharge	Sum	[64, 137]
	Time spent on Services	1. Throughput time (time in the system, Overall patient service Time, Mean Flow Time)		Avg	[18, 29, 41, 45, 47, 58, 64, 83, 85, 99, 113, 121, 124, 138, 141]
		Enter to ED	2. Ambulance response time	Sum	[118]
			3. Ambulance offload time	Sum	[114]
		Admission and Triage	4. Registration Time	Avg	[13, 64]
			5. Time in Triage	Avg	[13]
		Treatment	6. Patient treatment time	Sum	[13, 64, 99, 102]
			7. time with the doctor	Avg	[13, 64, 70]
			8.Stay > 3h. in bed	Cut of point	[1]
		Diagnosis	9. X-Ray Time	Sum	[107]
			10. Lab Time	Sum	[107]
			11.LAB TAT	Sum	[22]
		12. Service Level		Level	[31, 60]
		13. Elective Cancellation		Number	[5, 29, 64]
		14. Waiting Count		Avg, Number	[18, 25, 26, 36, 41, 50, 64, 65, 70, 71, 90, 112, 124, 130]

Table 6 presents the ED output indicators, with 16 leading indicators related to output indicators. The treated patient, identified as the most critical indicator among output indicators in the retrieved articles, is followed by the Throughput indicator.

**Table 6:** Output indicators of Emergency Department KPIs

Theme	Code	Consideration	References
Output indicators	1. Throughput	Avg	[5, 18, 22, 24, 40, 64, 73, 87, 141]
	2. Number of Diversions	Number/percentage in a month	[5, 22, 64]
	3. Diversion Time	Sum Overloading	[64, 67, 71, 89]
	4. Time TR* blocked for Contaminated patients	Number	[1, 132, 135]
	5. Time TR(WZ**) seized	Number	[1, 132, 135]
	6. Treated patients	Number	[1, 13, 20, 21, 23, 31, 35, 40, 41, 45, 54, 58, 63, 64, 66, 67, 69, 76, 78, 79, 84, 97, 98, 102, 115, 118, 125, 144]
	7. Patients served	Number	[18, 88, 130, 133]
	8. Wrongly discharge patient	Number	[23]
	9. Discharged patients	Number	[23–27, 142]
	10. Patients go out from ED in 12 h	Percent	[122]
	11. Patients’ disposition in 6 h	Percent	[122]
	12. Death	Number	[23]
	13. Lifesaving rate	Avg. decrease of	[18]
	14. Boarding Count	Number	[36, 71]
	15. Remaining Patient Care Load (RPCL)	Percent	[119]
	16. Companions of Patients	Number	[21, 63, 76]

*TR = Treatment Room

**WZ = Waiting Zone

Table 7 shows nine indicators related to cost and revenue ED KPIs. They were identified and classified into five main categories: Total Cost, Diagnostic Cost, Operational Cost, Overhead Cost, Revenue, and Budget. The cost indicator is the most popular among these indicators.

**Table 7:** Cost and revenue-based Emergency Department KPIs

Theme	Sub-theme	Code	References
Cost-based indicators	Total Cost	1. Cost	[5, 8, 18, 31, 34–38, 75, 83, 85, 94, 121, 129]
	Diagnostic Cost	2. Audiology Cost	[83]
		3. Radiology Cost	[83]
		4. Laboratory Cost	[83]
	Operational Cost	5. Operational Cost	[18, 39]
	Overhead Cost	6. Labor Cost	[18, 38, 39, 137]
		7. Medical Resource Wasted Cost (MWC)	[81]
	Revenue	8. Revenue-related measures	[5]
	Budget	9. Budget	[24]

In Table 8, we have presented a comprehensive analysis of 35 resource utilization and productivity indicators, meticulously categorized into four groups: Utilization, Efficiency, Productivity, and Quality. This comprehensive approach ensures that we cover all aspects of resource performance. Seven indicators were placed in the utilization category, four in the efficiency category, 12 in the productivity category, and 12 in the quality category. It is worth noting that Length of Stay (LOS), Leave Without Being Seen (LWBS), Resource Utilization, Human resource Utilization, and Equipment Utilization are the most important indicators related to Resource Utilization and Productivity, respectively.

**Table 8:** Resource utilization and productivity-related Emergency Department KPIs

Theme	Sub-theme	Code	`Considerations	References
Resource Utilization and Productivity-related indicators	Utilization	1. Resource Utilization	Percent	[8, 13, 18, 25, 29, 34, 36, 40, 63–65, 75, 85, 98, 112, 116, 122, 133]
		2. Location Utilization	Distances, Meters Emrg. Room, Treat. Room, Reception, Financial Department	[24, 73, 75, 110, 139]
		3. Human Resource Utilization	Percent Respiratory Therapists (RTs), Radiologist, Residents, Doctors, Nurses, Patients Access Representatives (PARs), Technicians, Registrar	[5, 13, 18, 24, 41, 45, 47, 56, 58, 61, 64, 73, 88, 91, 110, 121, 128, 130, 139, 140]
		4. Equipment Utilization	Percent Tube Stations, Bed, Radiology, Laboratory, Ambulance	[5, 13, 18, 24, 42, 45, 56, 75, 118, 121, 136, 139, 140]
		5. Scheduled Utilization	Sum	[25]
		6. Occupancy Level	Level	[29, 58, 64, 71, 116]
		7. Number busy	Number Time full (time), Time in use, Time starts use (time)	[1, 24, 25, 132, 135]
	Efficiency	8. Resource efficiency	Cut of Point, Qualitative	[7, 142]
		9. Service Efficiency	Cut of Point, Qualitative	[18]
		10. Layout efficiency	Avg. Distance (Move) Equipment, Nurse, Doctor, Patient	[13, 18]
		11. Turnaround Time	Sum Triage, Diagnostic, Registration	[13, 79, 130]
	Productivity	12. LOS	Sum, cut of point Expected Length Stay > 4h, stayed between 6 to 12 h, Stayed > 12 h, Stay < 6h	[1, 5, 7, 18, 22–24, 26, 27, 35, 36, 38, 40–44, 48, 49, 51, 52, 54, 56, 60, 63–73, 77, 78, 80, 81, 87, 93–96, 103–110, 112, 114, 116, 117, 119, 122, 125, 128, 129, 131, 132, 135, 139, 140, 142, 143]
		13. Human resource productivity	Ratio Patients: Nurse Ratio, Patients: Doctor Ratio	[13]
		14. Resource Productivity	Cut of Point, qualitative	[54]
		15. Crowding indicators	Percent	[1, 62, 94, 110, 117, 132, 134, 135]
		16. Real-Time Emergency Analysis of Demand Indicator (READI)	Percent	[123]
		17. Emergency Department Work Index (EDWIN)	Percent	[123]
		18. National Emergency Department Overcrowding Scale (NEDOCS)	Percent	[18, 42, 58, 123]
		19. Time of Peak	Sum	[1]
		20. Peak Crowding	Sum	[1, 132, 135]
		21. Overflow Probability	Percent	[64]
		22. Patient Los > 6 h	Percent/number	[13]
		23. Work Load	Avg	[131]
	Quality	24. Resource Requirement	Qualitative	[18]
		25. Adverse patients along the time	Avg. percentage	[62]
		26. Queues less than a target waiting time	Percent	[64]
		27. The proportion of patients meeting waiting time targets	Proportion	[64]
		28. Tardy patients	Percent	[87]
		29. Unsuccessful CPR	Percent	[122]
		30. LWBS	Number	[5, 8, 13, 18, 23, 26, 34, 39, 40, 45, 64, 66, 77, 109, 110, 122]
		31. Performance Provider	Qualitative	[55]
		32. Sigma Level	Level	[18]
		33. Job satisfaction	Qualitative	[37]
		34. Patient Satisfaction	Qualitative	[5, 36]
		35. Patient safety	Qualitative	[5]

Table 9 shows the growth in the use of KPIs of ED over time. As can be seen, only time-related indicators and human resource-related indicators were used in the period 1986–1996. With the entry into the period 1997–2006, the diversity of time-related indicators was increased, and LOS, physical space, and equipment and facilities indicators were also used. Since 2007, crowding-related indicators, quality indicators, output-related indicators, LWBS, and cost-related indicators have been added to the list of indicators used to measure ED performance. In the period 2017–2024, in addition to the indicators used so far and their increased diversity, indicators related to pandemics and patient demand have been used to evaluate ED performance during a pandemic. It is worth noting that the waiting time and LOS have been used almost throughout time.

**Table 9:** The usage of KPIs related to ED over the years

Year	KPIs
1986–1996	Waiting time to the doctor, Throughput time, Human Resource Utilization
1997–2006	LOS, waiting Time, waiting Count, wait time for each resource, waiting time in the queue, The time interval of the patient throughout the process, Throughput time, Human Resources, Facilities and equipment, Physical Spaces
2007–2016	Human Resources, Physical Spaces (ED), Facilities and equipment, waiting Time, waiting time to triage/Register, waiting time before admission to the consultation room, Waiting Time (triage to starting of visit), Waiting Time (from Arrival until evaluation by a practitioner), Wait for bed, waiting time for diagnostic/treatment, waiting time to the doctor, waiting to be seen (WTBS), waiting in nurse > 20 min, waiting time for test result, Boarding Time, waiting time to discharge, wait time for each resource, waiting time in the queue, waiting time in the waiting room, Buffer/wasting Time, The time interval of the patient throughout the process, The time between arrival and triage, Triage to disposal decision time, The time between triage and registration, Triage to Bedtime, DOT (difference between the starting time of triage to visit), Time from registration to ED physician Consult and to discharge, Door to bed, Time until first seen, Time to doctor, Door to doctor time, Doctor to Disposition, Time to treatment (TTT),Disposition to door out, First assessment to discharge, Registration Time, Time in Triage, Patient treatment time, time with the doctor, Stay > 3h. in bed, X-Ray Time, Lab Time, Throughput, Number of Diversions, Diversion Time, Companions of Patients, treated patients, patients served, Discharged patients, Cost, Audiology Cost, Radiology Cost, Laboratory Cost, Operational Cost, Labor Cost, Medical Resource Wasted Cost (MWC), Budget, Resource Utilization, Location Utilization, Human Resource Utilization, Equipment Utilization, Scheduled Utilization, Occupancy Level, Resource efficiency, Resource Productivity, Crowding indicators, National Emergency Department Overcrowding Scale (NEDOCS), Overflow Probability, LWBS, Performance Provider, LOS, Patient Satisfaction, tardy patients, Number busy, lifesaving rate, Sigma Level, adverse patients along the time, Occupancy Level, Service Level, Elective Cancellation, Layout efficiency, The proportion of patients, performance provider
2017–2024	waiting Time, waiting time to triage/Register, waiting time before admission to the consultation room, waiting Time (triage to starting of visit), Waiting Time (from Arrival until evaluation by a practitioner), Wait for bed, waiting time for diagnostic/treatment, waiting time to the doctor, waiting to be seen (WTBS), waiting in nurse > 20 min, waiting time for test result, Boarding Time, waiting time to discharge, wait time for each resource, waiting time in the queue, waiting time in the waiting room, Buffer/wasting Time, The time interval of the patient throughout the process, The time between arrival and triage, Triage to disposal decision time, The time between triage and registration, Triage to Bedtime, DOT (difference between the starting time of triage to visit), Time from registration to ED physician Consult and to discharge, Door to bed, Time until first seen, Time to doctor, Door to doctor time, Doctor to Disposition, Time to treatment (TTT), Disposition to door out, first assessment to discharge, Throughput time (time in the system, Overall patient service Time, Mean Flow Time), Ambulance response time, Ambulance offload time, Registration Time, Time in Triage, Patient treatment time, time with the doctor, stay > 3h. in bed, LAB TAT, Service Level, Elective Cancellation, Waiting Count, patients go out from ED in 12 h., patients’ disposition in 6 h., Death, lifesaving rate, Boarding Count, Remaining Patient Care Load (RPCL), Throughput, Time TR* blocked for Contaminated patients, Time TR(WZ**) seized, Revenue-related measures, Service Efficiency, Turnaround Time, Human resource productivity, Crowding indicators, Real-Time Emergency Analysis of Demand Indicator (READI), Emergency Department Work Index (EDWIN), National Emergency Department Overcrowding Scale (NEDOCS), Time of Peak, Peak Crowding, Patient LOS > 6Hrs, Work Load, Resource Requirement, adverse patients along the time, queues less than a target waiting time, The proportion of patients meeting waiting time targets, tardy patients, Sigma Level, Job satisfaction, Patient Satisfaction, Patient safety, Cost, LOS, discharged patients, Job satisfaction, Boarding Time, wrongly discharged patient, Death, X-Ray Time, LAB Time, Labor Cost, Remaining Patient Care Load (RPCL), unsuccessful CPR, patients’ disposition in 6 h, patients go out from ED in 12 h patients served, Number busy, Resource efficiency

## Discussion

The complexity and stochastic nature of the ED processes have made simulation a proper option in modeling an ED performance measurement. Therefore, simulation papers can consider a wide variety of KPIs and improvement choices [5]. The present study aims to identify the ED KPIs in simulation studies through a scoping review and classify them using the BSC framework to evaluate their performance in ED simulations.

Almasi (2021) et al., found 26 KPIs related to ED and divided them into five major categories (quality of care, patient flow, timeliness, cost, and resources) [15]. Núñez et al. (2018) proposed 75 KPIs in ED that have been classified into five main categories. They include quality (23 indicators), time (20 indicators), economy (15 indicators), capacity (11 indicators), and outcome (6 indicators) [16]. Ouda et al. (2023) introduced "Length of stay," "Wait time," "Door to the doctor," "Seen to the doctor," "Left without being seen," "Cost," and "Utilization or workload" as seven main categories of KPIs in ED [17]. Gul and Guneri (2015) present five main groups of KPIs [18]. Vanbrabant et al. showed that many simulation studies consider time-related indicators, and they reported that a mixture of KPIs is more significant [5]. It is similar to the current study's findings. The most significant number of KPIs related to processing time indicators (N = 46 KPIs).

Ismail et al. (2010) showed that utilizing simulation models with the BSC approach will assist in identifying ED bottlenecks. In the same way, it is caused to direct policymakers, administrators, and personnel to proper decisions and revised procedures. They have suggested "patients," "the process of ED," and "the development and training" as the main dimensions of BSC [13]. Safdari et al. (2014) concluded that "ED internal processes" and "timeliness and accessibility of care" are of the highest importance from the respondents' view [19]. The current study discusses the most important indicators in each dimension according to the BSC framework.

### Output/customer-related dimension

The 16 indicators (14%) were related to the output indicators. Most output indicators are related to the treated patients, throughput, discharged patients, diversion time, companion patients, and number of diversions, respectively. However, only one paper has considered death as an output indicator.

Atalan and Dönmez (2020) showed that the number of doctors is the most significant factor affecting the number of treated patients [20]. Taboada et al. (2011) reported that increasing the staff number and their experience led to growth in ED-treated patients [21]. In the present study, treated patients have been classified in the output category; however, Ismail et al. (2010) classified treated patients in the ED productivity category [13]. Finally, 28 papers of retrieved articles have discussed treated patients.

In 8% of the reviewed papers, patient throughput was pointed out, while Vanbrabant et al. (2019) indicated that 15% of reviewed articles considered patient throughput. Furthermore, they categorized it as productivity and utilization KPIs [5]. In contrast, the current study has categorized patient throughput as output KPIs. Although ED throughput is an important area, there are limited studies on this subject, and it should be investigated more in the future [22].

88% of ED patients will be discharged, and the rest will be hospitalized for treatment [23]. Early discharged patients can assist in decreasing the ED crowding problem. There are some solutions to discharge patients sooner. Using qualified staff and experienced physicians who can make prompt decisions and existing a holding area to admit patients earlier are appropriate strategies [48]. According to the reviewed papers, only five articles [23–26], and [27] used the discharged patient’s indicator.

### Time of process/internal process related dimension

Time of process indicators comprise a significant share of ED KPIs in the present study. Forty-one papers (38%) have referred to the 'waiting time' indicator. Also, 15 subcategories of 'waiting time' indicators (22 papers) have been identified. Long waiting time is an important issue that could adversely affect treatment procedures in the ED. Reducing waiting time can diminish crowding and bed occupancy time [16]. The findings of Farrahi (2019) showed that waiting time is the most influential performance measure in ED in a crisis [28]. Mohiuddin et al. (2017) reported that most of the reviewed studies (81%) pointed out waiting time as a performance measure, and 11 studies discussed only waiting time [29]. Wakai et al. (2013) have mentioned that focusing on time-related indicators in measuring ED performance is common and has some disadvantages. These indicators could not assist us in distinguishing the time spent on delivering care from the waiting time for the subsequent process, which refers to the next step in the patient's treatment journey. Moreover, by time-related indicators, the moving speed of patients through the ED has been considered more important than the quality of care provided [30].

### Cost and revenue/financial related dimension

The results show minimal use of cost and revenue-related indicators in simulation studies. Only 16 papers (15%) consider cost and revenue-related indicators to measure ED performance in simulation studies. It aligns with Vanbrabant et al. findings, which divided budget-related indicators into cost and revenue KPIs [5]. Gul and Guneri (2015) indicated that very few papers discuss managing ED costs as a KPI. Thus, they suggest measuring the cost of implemented scenarios to achieve improvement goals effectively [18]. Rashedi [31], Riyahifard [32], Sibuyeh [33], Gharahighehi [34], Hamza [35], Gharahi [8], Maleki [36], and Yousefinejad Atari [37] have evaluated the simulated scenarios cost. Guo et al. (2017) reported that growth in labor expenditure only sometimes causes ED efficiency improvement. Therefore, implementing a proper strategy for human resources is crucial. They indicated that human resources expenses in the examined hospital should be diminished [38]. Nahhas et al. (2017) mentioned that one of the simulation's aims is to balance operational costs [39].

### Resources (input)/growth and development related dimension

The 13 papers (12%) considered human resources to evaluate ED performance, and the authors classified this indicator in the resource utilization and productivity category. This finding aligns with the results from Vanbrabant et al. [5]. Nurses, physicians (33 papers), and official staff (15 papers) are the most prevailing human resources used in reviewed studies. Physicians and nurses are the resources affected by crowding, and there are often bottlenecks in the ED. So, many papers discussing human resources concentrated on physicians and nurses [5].

However, resource utilization indicators have rarely been used in simulation studies but are essential in measuring healthcare system performance [40]. In contrast, Samah et al. (2003) model showed that the main problems of ED would be solved through process revision, not adding new resources [41]. Maleki et al. (2014) used the utilization rate of resources [36]. Gharahi et al. (2014) considered human resources and equipment utilization KPIs [8]. Although equipment utilization is a significant performance indicator, it is discussed less in previous studies [42]. 9.5% of papers have discussed the equipment utilization to measure ED performance. Bed utilization and radiology equipment utilization are the popular indicators in papers. It is in line with Vanbrabant et al. [5].

### Management (vision, objectives, and strategies)/management-related dimension

The management dimension is the other performance measurement aspect based on BSC, which includes the productivity indicators category (efficiency and effectiveness), quality, and satisfaction indicators. Meanwhile, among productivity indicators, LOS is a crucial indicator in measuring ED performance.

The findings show that about 70% (74 papers) of the reviewed articles have pointed out the resource utilization and productivity indicators. LOS (56 papers) is the most replicated indicator among reviewed articles, followed by LWBS (16 papers), resource utilization (16 papers), human resource utilization (13 papers), and Equipment utilization (10 papers). Aroua and Abdulnour (2018) and Samaha et al. (2003) just used LOS to measure ED performance [41, 43]. Ghanes et al. (2015) investigated the relationship between staffing budget and LOS. They found that increasing the staff budget caused a decrease in LOS [44]. Gul & Gunri (2015) reported that the majority of ED simulation studies have concentrated on decreasing LOS and waiting time indicators [18], which is similar to the present study findings.

LWBS was identified as one of the management dimensions KPIs concerning quality. Oh et al. (2016) reported that 20% of patients who wait more than 30 min leave the ED without a physician being seen [45]. 15% of reviewed studies argued LWBS as a performance measure. Additionally, LWBS are signs of ED crowding. Some external factors such as proximity to health care facilities, demographic characteristics, and the number of patients according to triage level affect the LWBS indicator and make ED comparisons based on LWBS hard. Therefore, LWBS is rarely used as the only KPI in simulation studies. Vanbrabant et al. (2019) put LWBS in "proportion KPIs" [5], while the authors of this study have classified it as resource utilization and productivity indicators. Gharahi et al. (2014) suggested increasing inpatient and diagnostic department capacity to reduce LWBS indicators [8].

In the present study, Patient satisfaction indicators are qualitative and related to resource utilization and productivity. This classification is similar to that of Vanbrabant et al. (2019). They indicated that due to the difficulty of measuring quality indicators, only some operational studies have used them [5]. By contrast, Wakai et al. (2013) have classified ED patient satisfaction in the outcome indicator category [30]. Maleki et al. (2014) compared the ED simulated scenarios by the number of unsatisfied patients [36].

Considering all dimensions of BSC, the findings showed that LOS, waiting time, human resources, and treated patients are the most prevalent indicators of the present study. Shirazi (2016) presented waiting time, queue length, patient cycle time, and resource utilization as frequently used performance indicators in simulation studies [40]. Gul & Guneri (2015) have concluded that many studies discussed LOS, resource utilization, discharged patients, and financial indicators as main KPIs respectively [18]. Yousefi and Ferreira (2017) have introduced the number of patients who left without being seen, waiting times, length of stay, discharged patients, and time to a doctor as the most frequently used KPIs in the simulation studies [23]. Lotfi (2012) used KPIs such as waiting time, LOS, and the average number of treated patients to measure ED performance [46].

Finally, ED features could affect comparison and performance evaluation among EDs. The performance measurement status can be different according to the type of ED (teaching or clinical ED, specialized or general ED …), patient's characteristics, type of patients' triaging, ED personnel number, work hours, the existence of information system, and access to diagnostic departments. Therefore, these factors should be considered in ED KPIs comprehension and interpretation [19].

### Limitation

The current study has faced some limitations. Firstly, we only considered English and Persian studies, so some information in other languages might have been ignored. Secondly, ambiguity in the definitions of some indicators made it difficult to classify them into the appropriate categories. Moreover, some indicators could fall into more than one category simultaneously. We tried to solve this problem by discussing it with researchers. Thirdly, although this scoping review included a wide range of articles, it is possible that we missed some important articles due to the selection criteria or search limitations. Fourth, this study provides a general view of KPIs however, it can’t evaluate complex analyses such as cause-and-effect relationships between indicators.

## Conclusion

The findings showed that most simulation studies concentrated on time-related indicators of ED, which significantly contribute to ED performance measurement. In contrast, fewer studies have addressed the role of cost-related indicators, resource utilization, especially equipment utilization, input indicators, and output indicators. Also, due to the difficulty of measuring qualitative indicators, they have been used less in measuring ED performance. Therefore, it is necessary to consider qualitative and cost-related indicators in future studies. Furthermore, the most popular simulation methods utilized in the papers were DES, ABS, and hybrids, respectively. The difference between papers with the DES, ABS, and hybrid methods is considerable. Thus, it is suggested that more studies should be conducted using the ABS and hybrid methods. It is worth noting that the characteristics of ED should also be considered in the KPI selection to achieve a more accurate and correct performance measurement of ED.

## Supplementary Information

Supplementary Material 1: Details of search strategies

## Data Availability

This published article and its supplementary files include all data generated during this study.

## Abbreviations

EDs: Emergency Departments
KPIs: Key Performance Indicators
BSC: Balanced Scorecards
WT: Waiting Times
LOS: Length of Stay
LWBS: Leave Without Being Seen
