# Supplementary material for: Key performance indicators in emergency department simulation: a scoping review
Source: Scand J Trauma Resusc Emerg Med. 2025 Jan 30;33:15. doi: 10.1186/s13049-024-01318-7 (PMC11784001; doi:10.1186/s13049-024-01318-7)
Supplement: Supplementary file 1 — Supplementary Material 1: Details of search strategies [file 13049_2024_1318_MOESM1_ESM.docx]

**Search strategies based on Databases**

| **PUBMED** | **(((((((((((((((Emergency Hospital Service[Title/Abstract]) OR (Hospital Emergency Service*[Title/Abstract])) OR (Hospital Service Emergent[Title/Abstract])) OR (Emergency Unit*[Title/Abstract])) OR (Accident[Title/Abstract] OR (Emergency Department[Title/Abstract])) OR (Emergency Ward[Title/Abstract])) OR (Emergency Departments[Title/Abstract])) OR (Emergency Room*[Title/Abstract])) OR (emergency medical service*[Title/Abstract])) OR (hospital emergency department[Title/Abstract])) OR (emergency[Title/Abstract])) OR (Emergency Outpatients Unit*[Title/Abstract])) OR (Emergency Service, Hospital[MeSH Terms])) OR (emergency care system*[Title/Abstract])) OR (Emergency Medicine[MeSH Terms])) AND ((((((((((((((((((((((((((((((((Key performance indicate*[Title/Abstract]) OR (performance metric*[Title/Abstract])) OR (Assessment Process*[Title/Abstract])) OR (Process Assessment*[Title/Abstract])) OR (Process Assessment Health Care*[Title/Abstract])) OR (Process Measure*[Title/Abstract])) OR (Process Assessment, Health Care[MeSH Terms])) OR (performance improve*[Title/Abstract])) OR (process improve*[Title/Abstract])) OR (operational metric*[Title/Abstract])) OR (operational measure*[Title/Abstract])) OR (Workflows[Title/Abstract])) OR (Work Flow*[Title/Abstract])) OR (Workflow[MeSH Terms])) OR (operational improve*[Title/Abstract])) ) OR (Quality Improvements[Title/Abstract])) OR (Improvement*, Quality[Title/Abstract])) OR (Quality Improvement[MeSH Terms])) OR (Quality Indicator*, Healthcare[Title/Abstract])) OR (Healthcare Quality Indicator*[Title/Abstract])) OR (Indicator*, Healthcare Quality[Title/Abstract])) OR (Quality Indicators, Health Care[MeSH Terms])) OR (Organizational Efficiency[Title/Abstract])) OR (Efficiency, Administrative[Title/Abstract])) OR (Administrative Efficiency[Title/Abstract])) OR (Program Efficiency*[Title/Abstract])) OR (Efficiency, Program[Title/Abstract])) OR (Productivity, Organizational[Title/Abstract])) OR (Organizational Productivity[Title/Abstract])) OR (Efficiency, Organizational[MeSH Terms])) OR (patient flow*[Title/Abstract]))) AND ((((((((((((((((((((Analyses, Systems[Title/Abstract])) OR (Systems Analyses[Title/Abstract])) OR (Systems Oriented Approach*[Title/Abstract])) OR (Approach*, Systems Oriented[Title/Abstract])) OR (System Dynamics Analys*[Title/Abstract])) OR (Analys*, System Dynamics[Title/Abstract])) OR (Dynamics Analys*, System[Title/Abstract])) OR (Analysis, Systems[Title/Abstract])) OR (Systems Approach*[Title/Abstract])) OR (Approach*, Systems[Title/Abstract])) OR (Systems Thinking*[Title/Abstract])) OR (Thinking*, Systems[Title/Abstract])) OR (Systems Medicine*[Title/Abstract])) OR (Medicine*, Systems[Title/Abstract])) OR (Complexity Analysis[Title/Abstract])) OR (Analysis, Complexity[Title/Abstract])) OR (Complexity Analyses[Title/Abstract])) OR (Agent-Based Modeling[Title/Abstract])) OR (Agent Based Modeling[Title/Abstract]) OR (Agent-Based Modeling*[Title/Abstract]) OR (Modeling*, Agent-Based[Title/Abstract]) OR (Systems Analysis[MeSH Terms]) AND (Computer Simulations[Title/Abstract]) OR (Simulation*, Computer[Title/Abstract]) OR (Model*, Computer[Title/Abstract]) OR (Computerized Model*[Title/Abstract]) OR (Model, Computerized[Title/Abstract]) OR (Computer Model*[Title/Abstract]) OR (Computational Modelling[Title/Abstract]) OR (Modelling, Computational[Title/Abstract]) OR (Computational Modeling[Title/Abstract]) OR (Modeling, Computational[Title/Abstract]) OR (Computer Simulation[MeSH Terms]) OR (Simulation[Title/Abstract]) OR (agent-based simulate*[Title/Abstract]) OR (multi-agent system[Title/Abstract]))** |
| --- | --- |
| **SCOPUS** | TITLE-ABS ( "emergency hospital service" ) OR TITLE-ABS ( "hospital emergency service*" ) OR TITLE-ABS ( "hospital service emergent" ) OR TITLE-ABS ( "emergency unit*" ) OR TITLE-ABS ( accident ) OR TITLE-ABS ( "emergency department" ) OR TITLE-ABS ( "emergency ward" ) OR TITLE-ABS ( "emergency departments" ) OR TITLE-ABS ( "emergency room*" ) OR TITLE-ABS ( "emergency medical service*" ) OR TITLE-ABS ( "hospital emergency department" ) OR TITLE-ABS ( emergency ) OR TITLE-ABS ( "emergency outpatients unit*" ) OR TITLE-ABS ( "emergency service, hospital" ) OR TITLE-ABS ( "emergency care system*" ) OR TITLE-ABS ( "medicine, emergency" ) OR TITLE-ABS ( "emergency medicine" ) AND TITLE-ABS ( "Key performance indicate*" ) OR TITLE-ABS ( "performance metric*" ) OR TITLE-ABS ( "Assessment Process*" ) OR TITLE-ABS ( "Process Assessment*" ) OR TITLE-ABS ( "Process Assessment Health Care*" ) OR TITLE-ABS ( "Process Measure*" ) OR TITLE-ABS ( "Process Assessment, Health Care" ) OR TITLE-ABS ( "process improve*" ) OR TITLE-ABS ( "operational metric*" ) OR TITLE-ABS ( "operational measure*" ) OR TITLE-ABS ( workflows ) OR TITLE-ABS ( "Work Flow*" ) OR TITLE-ABS ( workflow ) OR TITLE-ABS ( "operational improve*" ) OR TITLE-ABS ( "Quality Improvement*" ) OR TITLE-ABS ( "Improvement*, Quality" ) OR TITLE-ABS ( "Quality Indicator*, Healthcare" ) OR TITLE-ABS ( "Healthcare Quality Indicator*" ) OR TITLE-ABS ( "Indicator*, Healthcare Quality" ) OR TITLE-ABS ( "Quality Indicators, Health Care" ) OR TITLE-ABS ( "Organizational Efficiency" ) OR TITLE-ABS ( "Efficiency, Administrative" ) OR TITLE-ABS ( "Administrative Efficiency" ) OR TITLE-ABS ( "Program Efficiency*" ) OR TITLE-ABS ( "Efficiency, Program" ) OR TITLE-ABS ( "Productivity, Organizational" ) OR TITLE-ABS ( "Organizational Productivity" ) OR TITLE-ABS ( "Efficiency, Organizational" ) OR TITLE-ABS ( "patient flow*" ) AND TITLE-ABS ( "Analyses, Systems" ) OR TITLE-ABS ( "Systems Analyses" ) OR TITLE-ABS ( "Systems Oriented Approach*" ) OR TITLE-ABS ( "Approach*, Systems Oriented" ) OR TITLE-ABS ( "System Dynamics Analys*" ) OR TITLE-ABS ( "Analys*, System Dynamics" ) OR TITLE-ABS ( "Dynamics Analys*, System" ) OR TITLE-ABS ( "Analysis, Systems" ) OR TITLE-ABS ( "Systems Approach*" ) OR TITLE-ABS ( "Approach*, Systems" ) OR TITLE-ABS ( "Systems Thinking*" ) OR TITLE-ABS ( "Thinking*, Systems" ) OR TITLE-ABS ( "Systems Medicine*" ) OR TITLE-ABS ( "Medicine*, Systems" ) OR TITLE-ABS ( "Complexity Analysis" ) OR TITLE-ABS ( "Analysis, Complexity" ) OR TITLE-ABS ( "Complexity Analyses" ) OR TITLE-ABS ( "Agent-Based Modeling" ) OR TITLE-ABS ( "Agent Based Modeling" ) OR TITLE-ABS ( "Agent-Based Modeling*" ) OR TITLE-ABS ( "Modeling*, Agent-Based" ) OR TITLE-ABS ( "Modeling*, Agent-Based" ) OR TITLE-ABS ( "Systems Analysis" ) OR TITLE-ABS ( "Computer Simulations" ) OR TITLE-ABS ( "Simulation*, Computer" ) OR TITLE-ABS ( "Model*, Computer" ) OR TITLE-ABS ( "Computerized Model*" ) OR TITLE-ABS ( "Model, Computerized" ) OR TITLE-ABS ( "Computer Model*" ) OR TITLE-ABS ( "Computational Modelling" ) OR TITLE-ABS ( "Modelling, Computational" ) OR TITLE-ABS ( "Computational Modeling" ) OR TITLE-ABS ( "Modeling, Computational" ) OR TITLE-ABS ( "Computer Simulation" ) OR TITLE-ABS ( simulation ) OR TITLE-ABS ( "agent-based simulate*" ) OR TITLE-ABS ( "multi-agent system" ) |
| **Web of Science** | #1 ((((((((((((((((TS=(Emergency Hospital Service)) OR TS=(Hospital Emergency Service*)) OR TS=(Hospital Service Emergent)) OR TS=(Emergency Unit*)) OR TS=(Accident)) OR TS=(Emergency Department)) OR TS=(Emergency Ward)) OR TS=(Emergency Departments)) OR TS=(Emergency Room*)) OR TS=(emergency medical service*)) OR TS=(hospital emergency department)) OR TS=(emergency)) OR TS=(Emergency Outpatients Unit*)) OR TS=(Emergency Service, Hospital)) OR TS=(emergency care system*)) OR TS=(Medicine, Emergency)) OR TS=(Emergency Medicine)  #2 ((((((((((((((((((((((((((((TS=("Key performance indicate*")) OR TS=("performance metric*")) OR TS=("Assessment Process*")) OR TS=("Process Assessment*")) OR TS=("Process Assessment Health Care*")) OR TS=("Process Measure*")) OR TS=("Process Assessment, Health Care")) OR TS=("process improve*")) OR TS=("operational metric*")) OR TS=("operational measure*")) OR TS=(Workflows)) OR TS=("Work Flow*")) OR TS=(Workflow)) OR TS=("operational improve*")) OR TS=("Quality Improvement*")) OR TS=("Improvement*, Quality")) OR TS=("Quality Indicator*, Healthcare")) OR TS=("Healthcare Quality Indicator*")) OR TS=("Indicator*, Healthcare Quality")) OR TS=("Quality Indicators, Health Care")) OR TS=("Organizational Efficiency")) OR TS=("Efficiency, Administrative")) OR TS=("Administrative Efficiency")) OR TS=("Program Efficiency*")) OR TS=("Efficiency, Program")) OR TS=("Productivity, Organizational")) OR TS=("Organizational Productivity")) OR TS=("Efficiency, Organizational")) OR TS=("patient flow*")  #3 (((((((((((((((((((((((((((((((((((TS=("Analyses, Systems")) OR TS=("Systems Analyses")) OR TS=("Systems Oriented Approach*")) OR TS=("Approach*, Systems Oriented")) OR TS=("System Dynamics Analys*")) OR TS=("Analys*, System Dynamics")) OR TS=("Dynamics Analys*, System")) OR TS=("Analysis, Systems")) OR TS=("Systems Approach*")) OR TS=("Approach*, Systems")) OR TS=("Systems Thinking*")) OR TS=("Thinking*, Systems")) OR TS=("Systems Medicine*")) OR TS=("Medicine*, Systems")) OR TS=("Complexity Analysis")) OR TS=("Analysis, Complexity") OR TS=("Complexity Analyses")) OR TS=("Agent-Based Modeling")) OR TS=("Agent Based Modeling")) OR TS=("Agent-Based Modeling*")) OR TS=("Modeling*, Agent-Based")) OR TS=("Systems Analysis")) OR TS=("Computer Simulations")) OR TS=("Simulation*, Computer")) OR TS=("Model*, Computer")) OR TS=("Computerized Model*")) OR TS=("Model, Computerized")) OR TS=("Computer Model*")) OR TS=("Computational Modelling")) OR TS=("Modelling, Computational")) OR TS=("Computational Modeling")) OR TS=("Modeling, Computational")) OR TS=("Computer Simulation")) OR TS=(Simulation)) OR TS=("agent-based simulate*")) OR TS=("multi-agent system"))  #1 AND #2 AND #3 |
| **EMBASE** | #1'emergency hospital service*':ab,ti OR 'hospital emergency service*':ab,ti OR 'hospital service emergent':ab,ti OR 'emergency unit*':ab,ti OR accident:ab,ti OR 'emergency department':ab,ti OR 'emergency ward':ab,ti OR 'emergency departments':ab,ti OR 'emergency room*':ab,ti OR 'emergency medical service*':ab,ti OR 'hospital emergency department':ab,ti OR emergency:ab,ti OR 'emergency service, hospital':ab,ti OR 'emergency care system*':ab,ti OR 'medicine, emergency':ab,ti OR 'emergency medicine':ab,ti OR 'emergency outpatients unit*':ab,ti  #2 'key performance indicate*':ab,ti OR 'performance metric*':ab,ti OR 'assessment process*':ab,ti OR 'process assessment*':ab,ti OR 'process assessment health care*':ab,ti OR 'process measure*':ab,ti OR 'process assessment, health care':ab,ti OR 'process improve*':ab,ti OR 'operational metric*':ab,ti OR 'operational measure*':ab,ti OR workflows:ab,ti OR 'work flow*':ab,ti OR workflow:ab,ti OR 'operational improve*':ab,ti OR 'quality improvement*':ab,ti OR 'improvement*, quality':ab,ti OR 'quality indicator*, healthcare':ab,ti OR 'healthcare quality indicator*':ab,ti OR 'indicator*, healthcare quality':ab,ti OR 'quality indicators, health care':ab,ti OR 'organizational efficiency':ab,ti OR 'efficiency, administrative':ab,ti OR 'administrative efficiency':ab,ti OR 'program efficiency*':ab,ti OR 'efficiency, program':ab,ti OR 'productivity, organizational':ab,ti OR 'organizational productivity':ab,ti OR 'efficiency, organizational':ab,ti OR 'patient flow*':ab,ti  #3 'analyses, systems':ab,ti OR 'systems analyses':ab,ti OR 'systems oriented approach*':ab,ti OR 'approach*, systems oriented':ab,ti OR 'system dynamics analys*':ab,ti OR 'analys*, system dynamics':ab,ti OR 'dynamics analys*, system':ab,ti OR 'analysis, systems':ab,ti OR 'systems approach*':ab,ti OR 'approach*, systems':ab,ti OR 'systems thinking*':ab,ti OR 'thinking*, systems':ab,ti OR 'systems medicine*':ab,ti OR 'medicine*, systems':ab,ti OR 'complexity analysis':ab,ti OR 'analysis, complexity':ab,ti OR 'complexity analyses':ab,ti OR 'agent-based modeling':ab,ti OR 'agent based modeling':ab,ti OR 'agent-based modeling*':ab,ti OR 'modeling*, agent-based':ab,ti OR 'systems analysis':ab,ti OR 'computer simulations':ab,ti OR 'simulation*, computer':ab,ti OR 'model*, computer':ab,ti OR 'computerized model*':ab,ti OR 'model, computerized':ab,ti OR 'computer model*':ab,ti OR 'computational modelling':ab,ti OR 'modelling, computational':ab,ti OR 'computational modeling':ab,ti OR 'modeling, computational':ab,ti OR 'computer simulation':ab,ti OR simulation:ab,ti OR 'agent-based simulate*':ab,ti OR 'multi-agent system':ab,ti  #1 AND #2 AND #3 |
